# Perceptual Gaps Between Clinicians and Technologists on Health Information Technology-Related Errors in Hospitals: Observational Study

**DOI:** 10.2196/21884

**Published:** 2021-02-05

**Authors:** Theophile Ndabu, Pavankumar Mulgund, Raj Sharman, Ranjit Singh

**Affiliations:** 1 Department of Management Science and Systems School of Management State University of New York at Buffalo Buffalo, NY United States; 2 School of Medicine and Biomedical Sciences State University of New York at Buffalo Buffalo, NY United States

**Keywords:** patient safety, medical errors, health information technology, sociotechnical framework, patient harm

## Abstract

**Background:**

Health information technology (HIT) has been widely adopted in hospital settings, contributing to improved patient safety. However, many types of medical errors attributable to information technology (IT) have negatively impacted patient safety. The continued occurrence of many errors is a reminder that HIT software testing and validation is not adequate in ensuring errorless software functioning within the health care organization.

**Objective:**

This pilot study aims to classify technology-related medical errors in a hospital setting using an expanded version of the sociotechnical framework to understand the significant differences in the perceptions of clinical and technology stakeholders regarding the potential causes of these errors. The paper also provides some recommendations to prevent future errors.

**Methods:**

Medical errors were collected from previous studies identified in leading health databases. From the main list, we selected errors that occurred in hospital settings. Semistructured interviews with 5 medical and 6 IT professionals were conducted to map the events on different dimensions of the expanded sociotechnical framework.

**Results:**

Of the 2319 identified publications, 36 were included in the review. Of the 67 errors collected, 12 occurred in hospital settings. The classification showed the “gulf” that exists between IT and medical professionals in their perspectives on the underlying causes of medical errors. IT experts consider technology as the source of most errors and suggest solutions that are mostly technical. However, clinicians assigned the source of errors within the people, process, and contextual dimensions. For example, for the error “Copied and pasted charting in the wrong window: Before, you could not easily get into someone else’s chart accidentally...because you would have to pull the chart and open it,” medical experts highlighted contextual issues, including the number of patients a health care provider sees in a short time frame, unfamiliarity with a new electronic medical record system, nurse transitions around the time of error, and confusion due to patients having the same name. They emphasized process controls, including failure modes, as a potential fix. Technology experts, in contrast, discussed the lack of notification, poor user interface, and lack of end-user training as critical factors for this error.

**Conclusions:**

Knowledge of the dimensions of the sociotechnical framework and their interplay with other dimensions can guide the choice of ways to address medical errors. These findings lead us to conclude that designers need not only a high degree of HIT know-how but also a strong understanding of the medical processes and contextual factors. Although software development teams have historically included clinicians as business analysts or subject matter experts to bridge the gap, development teams will be better served by more immersive exposure to clinical environments, leading to better software design and implementation, and ultimately to enhanced patient safety.

## Introduction

### Background

The widespread use of information technology (IT) has contributed to improved patient safety in the hospital setting [1-5]. However, many different kinds of medical errors attributable to the use of IT in health care have negatively impacted patient safety [6,7]. The number of patients who experience adverse events is estimated to be 40% of all patients who visit primary and ambulatory care [8]. These safety events may lead to an extended hospital stay, additional side effects, or distress and in some cases death. In addition to the loss of life and health impairment, the consequences of adverse events include increased financial costs to patients and the society at large [9].

In hospital settings, several benefits, including health care delivery improvement and reduction in medication errors, have been attained through the use of health information technology (HIT) [3]. However, new patient safety errors attributable to the use of HIT continue to be a significant issue [7]. For example, according to a recent study [10], in Pennsylvania alone, a total of 889 medication error reports listed HIT as a factor contributing to events submitted to the Pennsylvania Patient Safety Authority in the first 6 months of 2016. The study also shows that dose omission, wrong dosage, and extra dosage were the most commonly reported events. The most common HIT systems implicated in the events were the computerized prescriber order entry system, the pharmacy system, and the electronic medication administration record. Several government agencies and academic and clinical practitioner committees have been concerned about the unintended consequences of introducing IT in clinical environments. Several articles [9-11] report such adverse patient safety events related to HIT and emphasize the need for more cohesive HIT development processes to reduce the gulf of evaluation between medical and IT teams.

This pilot study seeks to classify patient safety events in hospital settings and to understand the differing perspectives of HIT designers and users concerning the potential causal factors of technology-related medical errors. In addition, the study suggests prescriptive measures to prevent reoccurrences of errors. Understanding the perspectives of both medical and IT stakeholders could help resolve the root causes of medical errors. The proposed classification could be used in facilitating medical and technology stakeholders in working together and working through different perspectives on the causes of HIT-related errors to identify likely solutions and ultimately design better HIT artifacts. To better understand the significant differences, we selected from our list of errors collected through the literature review, 12 archetype errors that occurred in a clinical setting, and examined them using the lens of sociotechnical theory from both clinical and IT systems perspectives. In the next section, we introduce the sociotechnical framework and present the proposed error classification. Following this, the Methods section details data collection and analysis. Subsequently, the results and discussion are presented before the Conclusions section.

### Sociotechnical Framework

The sociotechnical theory posits that organizational performance depends on the interactions between social and technical factors, grouped into 4 pillars: technology, process, people, and environment [12]. Prior research suggests that developing applications that cater to end-user needs requires designers and developers to understand the workflow structures, organizational culture, and environment in which these systems will operate [13]. Hence, patient safety improvement is contingent on the joint optimization of social and technical factors in the hospital setting.

This paper creates a more detailed taxonomy by adding subcomponents of the 4 central pillars to the sociotechnical framework [12,13]. The expanded taxonomy allows for a better classification of errors and the development of more precise solutions. Furthermore, we classify the errors in terms of the causes based on the feedback of medical experts and IT professionals. Using the results of this classification process, we provide more in-depth insights into the significant differences in medical and clinical staff members’ and IT professionals’ perceptions regarding these errors and offer a prescription to mitigate them.

Several studies have used the sociotechnical framework to examine several aspects of HIT implementation and use, including human-computer interaction [14], the impact of policy, infrastructure, and people on the quality of health information [15], ergonomic and macroergonomic aspects of health technologies [16-20], risk assessment of electronic medical record safety [18], and usability factors [14,18]. The sociotechnical framework has also been used to classify patient safety events [21-23]. However, these studies have classified errors on the sociotechnical framework’s high-level dimensions on which errors map the most (Table 1 shows a comparison of the 3 published papers closest to our efforts and details how this study is different). The sociotechnical framework suggests that multiple forces from multiple dimensions (and different hierarchical levels of a particular dimension) are at work when errors occur [24]. As patient safety events occur in a complex environment, there is a need for a classification that considers the impacts of multiple dimensions of the framework on each patient safety event’s occurrence. Table 1 provides a summary differentiating the studies closest to the work in this paper. These studies were included because the authors used the sociotechnical framework to classify medical errors [21,23] or HIT-related sentinel events [22].

**Table 1:** A comparison with previous studies based on the use of the sociotechnical framework

Studies (references)	Methodologies for error classification				
	Errors classified in 1 high-level dimension only—fitting one dimension excludes others	Errors classified in one dimension and its subdimensions only—fitting one dimension excludes others	Errors classified in multiple high-level dimensions	Classification based on multiple dimensions and their subcomponents	One error at a time
Safety huddles to proactively identify and address electronic health record safety [21]	✓^a^	✓	—^b^	✓	✓
Contribution of sociotechnical factors to health information technology–related sentinel events [22]	✓	—	—	—	—
Exploring the sociotechnical intersection of patient safety and electronic health record implementation [23]	✓	—	—	—	—
This study	—	—	✓	✓	✓

^a^Methodology applicable to the study.

^b^Methodology not applicable to the study.

**Figure 1:**
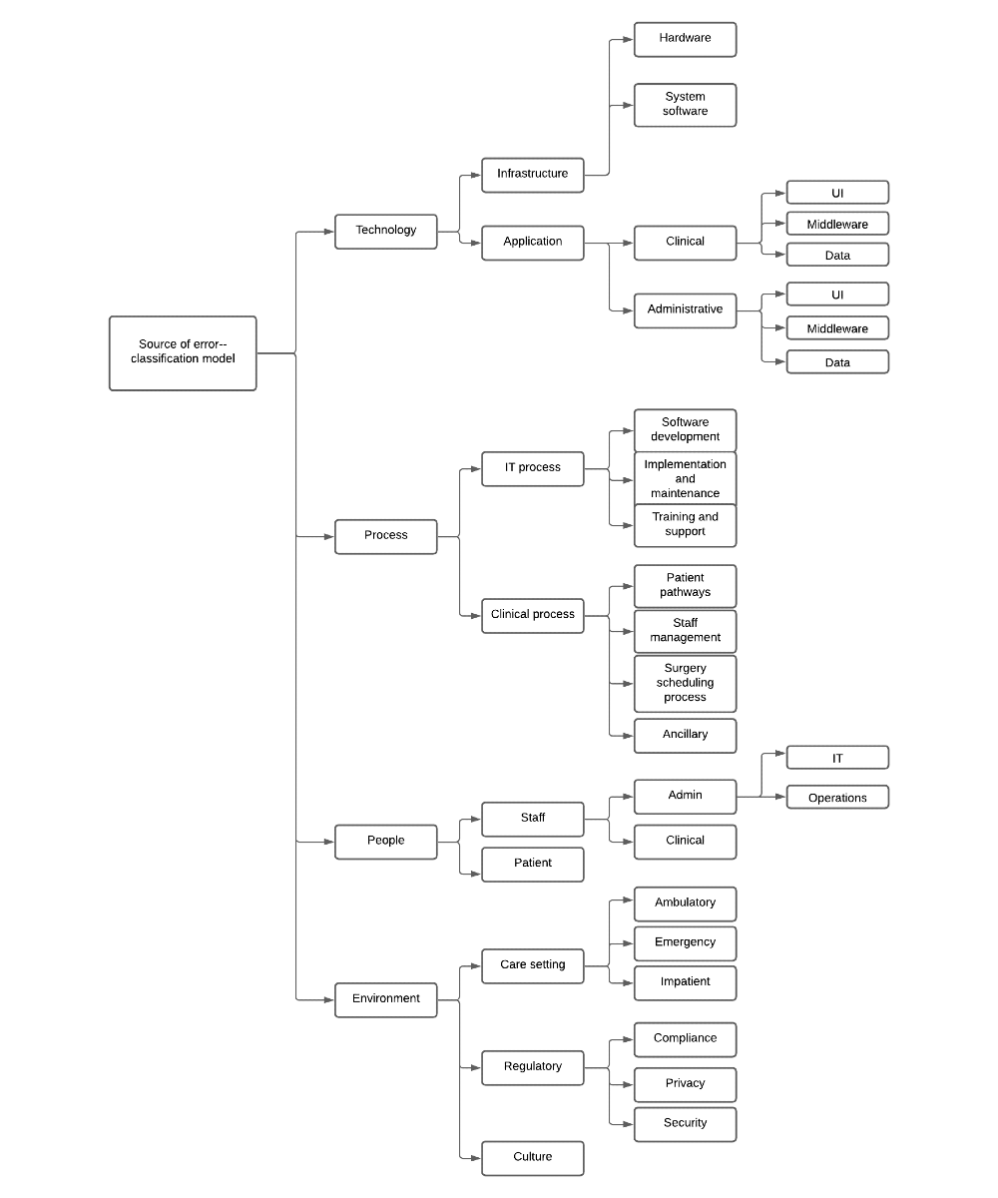
Error classification model. UI: user interface.

Medical error classifications have been developed using other approaches. The System Theoretic Accidents Models and Process framework has been used to classify medical errors in 3 broad categories: feedback, control action, and knowledge errors [25]. The Human Factors Classification Framework [26] has been adapted to health care to classify medical errors in 5 categories: decision errors, skill-based errors, perceptual errors, routine violations, and exceptional violations [27,28]. Other studies have developed taxonomies without the use of a particular framework [29-31]. Prior studies have not applied the sociotechnical framework on medical errors with the intent of exploring the root causes and potential avenues through which the errors can be fixed. Furthermore, the dimensions of sociotechnical frameworks described in the extant research literature have not considered the emergence of new technologies such as cloud computing, n-tier architectures, and new management paradigms, including DevOps and microservices architecture. We adapted and extended the sociotechnical framework with additional dimensions that reflect new trends in IT. A group of expert researchers in information systems and sociotechnical theory reviewed this model [32]. Feedback from these experts was incorporated to refine the classification model, which is presented in Figure 1.

### Proposed Classification

Sociotechnical theory emphasizes the interplay of the social and technical aspects of adopting and using technology [17,18,33]. The theory hinges on four basic constructs (technology, people, process, and environment) and the interaction between these constructs. In the expanded version of the sociotechnical framework, we detail the components of the technology dimension to include the IT infrastructure, which in turn comprises hardware, software, and apps. These also include emerging technologies, such as cloud computing, the internet of things, mobile apps, and the use of artificial intelligence, predictive and prescriptive analytics, and robotics. The technology dimension can also be partitioned based on the type of use, broadly classified as either administrative (including administrative IT and resource scheduling) or clinical. The need to investigate at this level of detail stems from the fact that the type of interaction varies based on the interacting subcomponents. Furthermore, the app layers can be viewed as comprising the user interface, middleware (including the logic layer), backend (including the logic layer), and data.

The process dimension includes administrative and clinical workflows. Administrative workflows related to IT include the collection, storage, processing, and presentation of information for more effective resource management, such as clinical and IT staff management, operating room scheduling, risk and safety management, billing and facility management, and inventory management to ensure the business management of the hospitals. The subdimensions of IT processes are software development, HIT implementation and maintenance, and training and support. Clinical processes include patient record management, clinical pathways, patient bed assignment, and physician notes. Some processes are both clinical and administrative; these include the inventory management of drugs and clinical supplies, surgery room and equipment scheduling, and patient discharge management. Processes in health care settings allow all stakeholders to perform tasks in a predetermined manner to obtain successful outcomes [24,34,35]. Patient safety errors manifest when there is a misalignment between the elements of IT and clinical processes.

The people dimension includes patients, clinical staff, and administrative staff. People interact with each other and with the technology available to them. The hospital employee space consists of providers with different competencies and clinical authorities and administrative staff with priorities that are often very different from those of clinical providers. Several examples are worth mentioning here. First, clinical staff members prioritize patients' clinical health, whereas IT personnel are more concerned with the processes involved in health care. Inconsistencies in their priorities often lead to errors. As people interact with the entire work system, a mismatch between people and any other components increases the risk of harm to patients. Human errors are also a threat to patient safety [36]. Therefore, it is essential to build user interfaces and systems that consider the priorities and goals of the different types of users of the system, and these goals go beyond the purely functional and technical requirements of the job.

The environment consists of the care setting (eg, ambulatory, emergency, and in-patient), regulatory (eg, compliance, privacy, and security related), and culture. Culture stems from management style, organizational policy, and other systemic factors. Furthermore, different types of employees prioritize different goals, and conflicts in achieving these goals are often manifest in the building, implementation, and functioning of systems. Patients receiving services are external to the health care organization. To ensure more effective health care service provisioning, patient participation in the process is very important. In some areas, tasks must be performed by patients away from the health care organization. Contextual environments and skills to perform the required tasks differ from those of health care providers [33,35]. Regulations can also have a constraining effect on the error-free functioning of all subsystems. A thorough classification of patient safety events should consider specific areas of interaction between the environment dimension and all other dimensions. We use this expanded classification model to understand the gap in the mental models of clinical staff and technology professionals regarding the root cause of errors and how they should be addressed. We articulate our research design in the next section.

## Methods

### Research Design

**Figure 2:**
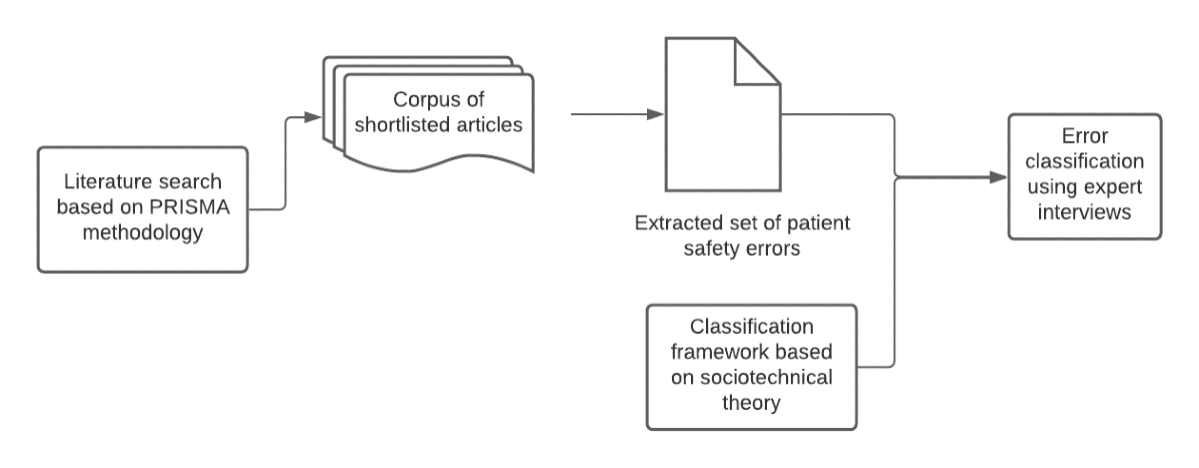
Research flow.

The research design is comprised of 2 significant steps: developing a shortlisted set of IT-related patient safety issues and the classification of the root causes of medical errors with the sociotechnical lens using expert interviews. Figure 2 depicts the flow of the study.

### Error Collection Using Literature Review

In this study, we first developed an extended sociotechnical framework that includes a finer level of granularity. Next, we systematically reviewed the literature on patient safety and medical errors from Ovid-MEDLINE, Embase, and Web of Science, which are leading medical databases in addition to Google Scholar. The systematic review process shown in Figure 2 aligns with commonly used steps of the PRISMA (Preferred Reporting Items for Systematic Reviews and Meta-Analyses) guidelines [37], as depicted by several exemplar papers [38-40]. The searches were performed using the following search terms: (“Patient Safety” OR “Medical”) AND (“issue” OR “error”) AND (“health information technology” OR “information technology”). Initially, the title, abstract, and index terms were used to screen published journal papers, conference papers, proceedings, case studies, and book chapters. We also used ancestral search to locate potentially relevant articles. Subsequently, the shortlisted papers were reviewed entirely. Two reviewers performed the screening independently. The reviewers met regularly to discuss the inclusion of the studies. A third reviewer was consulted when there was a discrepancy. Interrater reliability indicated a high agreement (Cohen κ value of 0.95).

Inclusion criteria included studies that addressed patient safety by identifying specific issues that occurred in health care settings and linked these errors to HIT. Furthermore, we excluded studies that were not available as the full text in the final search; were not in English; or were reports, abstracts only, letters, or commentaries.

### Expert Interviews

An invitation email to participate in the study was sent to the alumni of the University at Buffalo. The email contained the eligibility criteria consisting of ≥5 years of HIT experience and at least 1 IT-related professional certification. A separate invitation email mentioning the selection criteria was sent to medical experts through the Office of Business Coordination at the University at Buffalo. A minimum experience of 5 years working as a medical doctor or as a registered nurse was required to qualify for the interview. All participants who responded met the selection criteria and were included in the study.

To better understand the perspectives of different stakeholders, we conducted multiple semistructured interviews [41] with different stakeholders, namely 6 IT and 5 medical experts to map the errors on the different dimensions of the expanded sociotechnical framework. Experts could map an error on multiple (or on all) subdomains of the sociotechnical framework to show the different sociotechnical factors that could contribute to the error. The purpose of accounting for the different perspectives was to understand how each group understood the predicates of the problem and allow us to reflect on how best the error could be addressed. Interviews were selected based on their domain experience, education, and industry certifications. The IT experts, who were recruited from the alumni list of the State University of New York at Buffalo, were software development professionals with a master’s degree and IT professional certifications, such as the certified scrum master, the health level 7 control specialists, and the project management professional certifications. The minimum work experience cutoff for IT experts was 5 years for HIT in addition to possessing at least one IT-related professional certification.

IT experts who were interviewed had extensive IT experience (mean 10.33, SD 1.11 years) with significant HIT experience (mean 8.83, SD 2.03 years; Multimedia Appendix 1 uploaded as a supplementary file for brief profiles of IT interviewees). The medical experts interviewed were physicians and registered nurses with broad primary care experience from working with multiple health care institutions across the United States and Canada. They are all currently working with hospitals and institutions affiliated with the university at Buffalo (Multimedia Appendix 2). Medical experts had a mean experience of 16.6 (SD 7.33) years. The minimum and maximum numbers of years of HIT experience for IT experts were 5 and 12, respectively. The work experience of medical experts varied from 8 to 27 years. The questionnaire and interview process are detailed in Multimedia Appendix 3. Experts were asked to provide their opinions on why the selected errors (Multimedia Appendix 4 [42-48]) occurred and how the errors could be prevented. The extensive experience of both IT and medical experts in their respective domains qualifies them to map medical errors on the sociotechnical framework. The study was approved in November 2019 (IRB# STUDY00003838).

## Results

### Search Results

**Figure 3:**
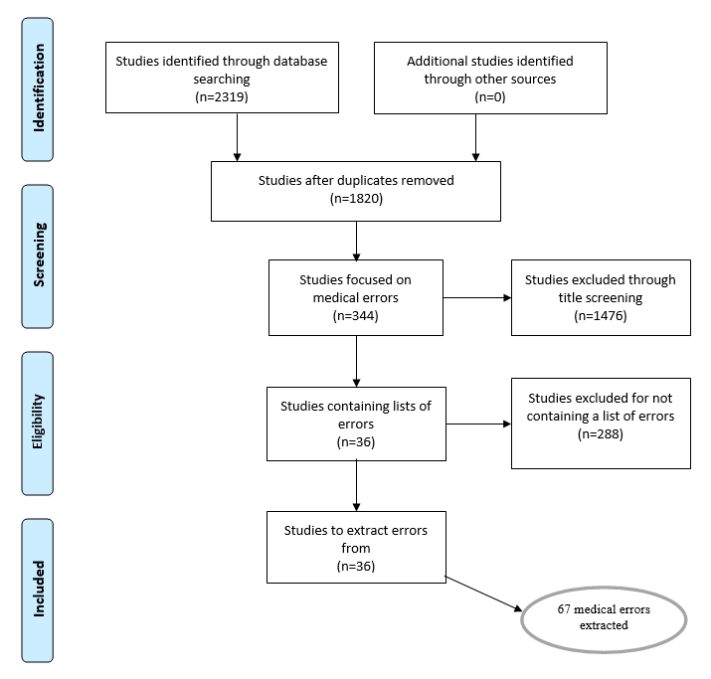
Data collection method.

The literature search resulted in 344 articles, 141 of which were duplicates. After removing articles based on their content, we retained 36 articles [10,28,42-47,49-76] that met the 2 criteria set for the study. We then extracted 67 unique patient safety events from the articles in which 12 specific issues related to IT use in the hospital setting were shortlisted. The process followed the PRISMA methodology [37] as detailed in Figure 3. The remaining errors occurred outside a health care setting and were excluded from the study. The error description includes the error context in the literature review format commonly known as problems, interventions, comparisons, and outcomes model [37]. The articles describing the errors contained a clear purpose, literature review, research methodology, results, and conclusions.

### Study Characteristics and Error Classification

In this study, experts categorized errors based on their opinion of where the source of the error lies. Experts were provided with the definitions of the elements of the framework and were informed that an error could result from multiple sources. They were asked to map each error at the lowest level of one or multiple dimensions of the sociotechnical framework. The authors then interacted with the experts to understand the reasons behind their mapping selection. The interactions included questions related to suggestions on the best way to address the problems and prevent them from occurring. In line with extant literature on data analysis in qualitative research coding [77,78], expert interviews were subsequently deconstructed into keywords and phrases and then grouped into ideas and concepts. The output of the analysis is summarized in the “key observations” below, for example, in Error 1: “Copied and pasted charting in the wrong window: Before, you could not easily get into someone else's chart accidentally...because you would have to pull the chart and open it.”

Medical experts highlighted several contextual issues, such as the number of patients a health care provider is set to see in a short time frame, unfamiliarity with a new electronic medical record system, nurse transitions around the time of the error, and confusion due to patients having the same name. They emphasized process controls, including failure modes, as a potential fix. The technology experts discussed the lack of notification, poor user interface, and lack of end-user training as critical factors in this error. Error 2: “Incompatible data standards across multiple mobile applications led to the missing of vital data fields, which led to information loss.”

Like the first sample, medical experts attributed this error to system software–related interoperability issues. They also highlighted several changes in the International Classification of Diseases (ICD) during the transition from ICD 9 to ICD 10 as an example of a situation that could lead to errors. Technology experts, however, emphasized data formats, data transfer protocols, and service-orientated architecture as potential causes of errors.

Although we have detailed 2 instances here, the experts reviewed all 12 errors and identified the most likely set of possible dimensions to which the errors could be attributed. The sample errors used in the study are presented in Multimedia Appendix 2, and the results of analyzing these data are presented in Table 2, followed by several key observations.

**Table 2:** Classification by medical and IT experts.

Errors	Classification by medical experts	Classification by IT^a^ experts
Nurse was supposed to enter a prescription for Amoxicillin 250 mg PO q8h×7 days (21 dispensed). However, the nurse failed to change the default dosage amount and dispensed too much medication (30 dispensed)	UI^b^-clinical app implementation and maintenance Clinical staff	UI-clinical app software development Clinical staff
Copied and pasted charting in the wrong window: “Before, you could not easily get into someone else’s chart accidentally...because you would have to pull the chart and open it”	Clinical staff Clinical app Training	Clinical staff In-patient
In general practice ward, the doctor consulted a patient with another patient’s records and prescribed medications according to the wrong records. The patient died the same day of taking it. No further details were available	Clinical staff Clinical UI Clinical middleware	Clinical UI Implementation and maintenance Staff-admin (IT)
The receptionist intended to alert the general practitioner via the practice software about a patient with chest pain but instead sent the message to himself. The patient later died from a myocardial infarction	UI-patient pathways Clinical staff	UI-clinical app software development Training and support Patient pathways
A patient received only half of their usual quantity of blood pressure medication because a repeat prescription for the medication did not transfer to a new software system when the patient's historical records were migrated. Because they did not have enough medication the patient tried to stretch out the old dose by taking the medication on alternate days. The patient had a stroke but made a full recovery.	Software-systems Patient pathways Patient	Data-clinical Software development Implementation and maintenance
A child had a full body x-ray. Some of the images went missing from the archival system where they were digitized. The x-ray was repeated to acquire the missing images, re-exposing the child to high levels of radiation	Software-systems Patient pathways	Data-clinical
A compound in high demand such as Rifampicin was not listed in the computerized physician order entry system. The consequence was that the physician could not order rifampicin.	Data-clinical Ancillary In-patient Culture	Data-clinical Software development Staff-admin (operations) Culture
When an update is made to the frequency field on an existing prescription, the frequency schedule ID is not simultaneously updated on new orders sent to the pharmacy via (application)	Software-development Clinical-people Software-systems	Data-clinical Staff-admin (IT) Software-systems
Monitoring and Eavesdropping on Patient Vital Signs by hacking into the packet transfer from an internet of things device to the central system	Middleware Maintenance People-staff (operations) Compliance Security	System software Data-clinical Software development Compliance Security
Vulnerabilities of the hospital’s IOT devices were exploited to initiate a denial-of-service attack to bring down hospital’s servers which disrupted normal functioning	Hardware Software IT implementation Compliance Security	Compliance Security
Use of portable devices that are not password protected makes the patient record vulnerable to the invasion of privacy	Data-clinical Software-development Maintenance Compliance Privacy	System software Software-development Security
Incompatible data standards across multiple mobile applications led to the missing of vital data fields which led to information loss	Software-systems Software-development	Data-clinical

^a^IT: information technology.

^b^UI: user interface.

## Discussion

### Principal Findings

Some of the crucial observations include (1) The identified potential sources of the errors and solution areas differed considerably between clinicians and IT specialists; (2) both groups identified multiple factors as potential causes of the errors; (3) the clinicians often focused on postproduction (eg, implementation, maintenance, training, context, and the way the application is used) issues as causal factors; (4) IT experts focused on software functionality, software development, and technical implementation issues as causal factors; (5) on most occasions when IT experts identified an issue as a “data” problem, clinicians seemed to think that the problem lay elsewhere, including the software system, software development, or patient pathways; (6) both groups seem to be congruent with the issues of compliance and security; and (7) IT experts rarely identified clinical pathways or workflows as an issue.

The classification of the identified medical errors using the framework is presented in Table 2. The continued occurrence of many errors is a reminder that current HIT software testing and validation do not seem adequate in terms of ensuring the functioning of the software within the health care organization. The attribution of the errors to different aspects of the sociotechnical framework by clinicians and IT professionals informs us that technologists and clinicians generally differ in their perspectives on factors that impact IT-related safety events. Software experts are often not acclimatized to the environment in which HIT software and tools are used, which could be a cause to the problem.

Although IT and medical experts’ perceptions are similar in security and privacy, IT specialists often tend to assume that the issues are either software or hardware or user interface related. In contrast, clinicians tend to consider environmental, contextual, and process factors as contributors to patient safety events. The benefit of such a classification suggests that designers and developers who fix the errors consider the artifact's environment and the people using the artifact. A key realization is that such errors will continue to occur if health IT system developers do not fully grasp the importance of technology functioning in an environment of care delivery where the patient needs are paramount.

A careful review of the IT experts’ classification of errors highlights the view that IT experts consider technology as the source of most errors and suggest solutions that are mostly technical. The IT experts highlighted the software systems and development as the top 2 sources of most errors. Similarly, the suggestions of potential fixes mostly revolve around the software. However, a common refrain that accompanied their answers was, “The doctor should double-check...” In contrast, clinicians tended to assign the source of errors within the people, process, and contextual (environmental) dimensions for the most part.

The difference in perspective could be explained by the fact that clinicians tend to deal with the system after implementation. In contrast, IT experts tend to look at the same problem from an IT development perspective. For example, for “Error 1,” for which IT experts were asked how they would prevent a doctor from using the wrong chart when he had multiple charts open, the answer was always to restrict access to 1 open chart at a time. However, clinicians prefer having multiple windows open so that they can quickly consult with multiple patients in different rooms without having to close out and reopen a chart. For them, the issue is, “How easy is it for a physician to realize the mistake,” and “Physicians should still be able to open multiple charts.” The differing perspectives between designers and developers of the technology and its users can contribute to medical errors.

The development teams of clinical applications typically include clinicians who provide domain expertise. However, our study indicates that this may not be sufficient as IT experts do not fully grasp the clinical environment and how workloads and other patient-related variabilities impact the use of the software. Therefore, as a future investigation, we suggest that software companies immerse developers in clinical environments for a short period, so that the understanding of the environment is built into their psyche and translates into a more robust design.

HIT systems can be made less error prone if programmers and systems developers understand the health care organization's operating environment. Current systems do not have fail-safe mechanisms that could prevent some of the errors. For example, consider the documented error, “the nurse was supposed to enter a prescription...the nurse failed to change the default amount and dispensed too much medication”; from a software perspective, better checks and warnings can be developed. In this specific instance, a system challenge asking the nurse to review the dosing amount could have prevented the problem. From a process perspective, nurses could be trained to reexamine the dosage. Creating a poka-yoke (like a check-off box for dose amount) would force nurses to check the dosing before refilling the prescriptions. As the clinical experts and IT experts suggested slightly different predicates for the error, a solution that addresses the issue from both technical and from a process and workforce training perspective would provide multiple layers of defense against such failures. The different views expressed by IT and clinical experts can be used to create technical and process solutions so that there is a more robust defense against these types of errors.

### Limitations and Future Studies

The results of this study should be interpreted cautiously, as there are several limitations to this study. The first shortcoming is related to the smaller number of participants interviewed in this study. Only 11 interviews comprising 5 medical providers and 6 HIT professionals were conducted. Therefore, this study should be considered a pilot study suggesting the differences in the mental models of the clinical and technical staff, which potentially leads to ineffective systems analysis and ultimately manifests as errors in practice. In addition, both IT and medical experts have, for the most part, acquired their education and expertise at affiliated institutions in the Northeast of the United States. Future studies should examine the hypothesis that medical experts are more likely to attribute medical errors to contextual factors, whereas IT experts on technical factors use a nationally representative sample.

Second, we shortlisted 12 unique errors that occurred in a hospital setting; the findings of this study cannot be generalized beyond that context. Furthermore, we extracted the errors used in this study from articles written in the English language. Future studies could examine errors that occurred in medical homes, patients’ homes, or other nonhospital settings or include studies written in other languages.

Third, the study did not examine errors that were discovered by HIT users before the occurrence of a patient safety event. Future studies should examine near-miss errors to determine their potential root causes and fixes using the lens of sociotechnical theory.

### Conclusions

This study classifies medical errors gathered from extant literature based on an expanded sociotechnical framework. Interviews from health care and IT experts reveal differing perspectives on why medical errors occur in clinical settings. Health care experts were more likely to attribute the source of an error to the implementation and use of an IT tool, whereas IT experts were likely to identify software design and functionality as causal factors of medical errors. From the results of this study, we offer several error-prevention prescriptions that can be tested in future research. First, IT experts should observe the functioning of HIT postimplementation and collect metrics related to its impact on (1) physician consultation time, (2) physician efficiency, (3) patient-physician relationship, (4) training needs, and (5) how the software fits into the workflow and culture of the organization. Software developers should be trained to be sensitive to the provider and patient needs because their lack of exposure to postproduction issues and usage contexts leads to the development of applications that do not cater to all user situations. Understanding these situations may lead to building software constraints and improved user training. Although software development teams have historically included clinicians as business analysts or subject matter experts to bridge the gap, development teams will be better served by more immersive training and exposure to clinical environments, leading to better software design and software implementation strategies.

## Appendix

Multimedia Appendix 1 Interviewees—information technology experts.

Multimedia Appendix 2 Interviewees—medical experts.

Multimedia Appendix 3 Interview process and questionnaire.

Multimedia Appendix 4 List of errors.

## Abbreviations

HIT: health information technology
ICD: International Classification of Diseases
IOT: Internet of Things
PRISMA: Preferred Reporting Items for Systematic Reviews and Meta-Analyses
UI: user interface
