# Supplementary material for: Perceptual Gaps Between Clinicians and Technologists on Health Information Technology-Related Errors in Hospitals: Observational Study
Source: JMIR Hum Factors. 2021 Feb 5;8(1):e21884. doi: 10.2196/21884 (PMC7971770; doi:10.2196/21884)
Supplement: Multimedia Appendix 1 [file humanfactors_v8i1e21884_app1.docx]

## Interviewees – IT professionals

1

Informant #1 is a Senior Product Manager with 9 years of clinical IT products. The Informant is currently employed with a Web-based electronic health record company headquartered on the West Coast of the United States and is in charge of the EMR and CPOE integrations. Informant #1 holds multiple certifications including the Certified Scrum Master (CSM), the Certified Scrum Product Owner (CSPO), the Certified SAFe 4 Agilist certifications.

2

Informant #2 is a Senior Product Manager with 11 years of health IT experience. The informant is employed with an American multinational health care services company headquartered in the Midwest of the USA and is in charge of IT applications responsible for prescription management and medication order fulfillment. The informant holds the Certified Scrum Master (CSM) certification.

3

Informant #3 is a Lead Application Developer (Full-Stack) at a Teaching hospital of a university in the Northeast of the United States. Informant #3 has 11 years in Health IT at the current employer and is currently in charge of application integration. The informant holds several health IT certifications including the Certified Scrum Master (CSM), and the Health Level 7 (HL7) Control Specialists certifications.

4

Informant #4 is currently a Senior Systems analyst at a hospital IT department in the Northeast of the USA. The informant has 12 years of total IT experience of which 8 are in Health IT at the current employer. The informant holds the Certified Scrum Master (CSM) certification.

5

Informant #5 is a senior analyst at a global consulting company headquartered in the Northeast of the United States. Informant #5 has 9 years in IT of which 5 years of health IT experience at a hospital in the Midwest. Informant #5 holds the Certified Scrum Master (CSM) and the Certified Professional in Health Information Systems (CPHMIS) certification.

6

Informant #6 is an Associate with a global consulting company headquartered in the Northeast of the USA. Informant #6 has 10 years of IT experience of which 9 in healthcare information systems consulting. Informant certifications: Certified Scrum Master (CSM) and HL 7 certifications.
