# Supplementary material for: Perceptual Gaps Between Clinicians and Technologists on Health Information Technology-Related Errors in Hospitals: Observational Study
Source: JMIR Hum Factors. 2021 Feb 5;8(1):e21884. doi: 10.2196/21884 (PMC7971770; doi:10.2196/21884)
Supplement: Multimedia Appendix 2 [file humanfactors_v8i1e21884_app2.docx]

## Interviewees – Medical professionals

1

Informant #1 is a medical doctor (MD) with ample administrative and informatics experience (over 15 years). Informant has served in multiple leadership roles at multiple institutions in the Northeast of the USA.

2

Informant #2 is a primary care physician with over 10 years of experience working in multiple capacities including administrative and technology-oriented roles at multiple institutions and in multiple cities in the Northeast of the USA.

3

Informant #3 is a nurse instructor with over 10 years of oncological nursing in the Northeast. The informant has trained and mentored fellow nurses on the use of EMRs. The informant is a Registered Nurse (RN).

4

Informant #4 is a Practicing Nurse with over 9 years of experience in the emergency care unit at a Community Healthcare Center in the Northeast. The informant is a Registered Nurse (RN).

5

Informant #5 works for a Center for Behavioral Health Services and is responsible for counseling and treating patients. Informant #5 is a medical doctor (MD) with over 7 years of practice experience.
