# Supplementary material for: Perceptual Gaps Between Clinicians and Technologists on Health Information Technology-Related Errors in Hospitals: Observational Study
Source: JMIR Hum Factors. 2021 Feb 5;8(1):e21884. doi: 10.2196/21884 (PMC7971770; doi:10.2196/21884)
Supplement: Multimedia Appendix 3 [file humanfactors_v8i1e21884_app3.docx]

Appendix- A

**Semi-structured Interview Process & Questionnaire.**

In order to conduct the semi-structured interview, we adhered to the following process and adapted the questions slightly to ensure it sounded appropriate for each group (IT experts and clinicians). We also ensured that our questions did not bias the respondents in any way. As part of developing the script, we conducted a pilot test with an expert from each team. This enabled us to make our processes clearer and our questioning unbiased.

The Interview process and questionnaire are shown below:

Step 1. Project one error at a time on a PowerPoint slide

Step 2. Provide details of the error as described in the literature

Step 3. Read one question a time from the list below. Move to the next question after all participants have answered the question by slotting where the error fits on the sociotechnical framework.

Questionnaire:

- Is the medical error clear to you?
- Do you need additional information to understand the context?
- List 3 most likely reasons why you think the error occurred?
- How can such an error be prevented?

Transcription:

A team of 3 researchers interviewed the experts. While the facilitator conducted the interview loosely following the interview guide, the other two researchers independently took notes. The interviews were also audio recorded and transcribed, and these documents were used to perform data analysis. In order to avoid potential research bias, researchers who were not involved in the interview performed the data analysis.
